# Integrated analysis of enzymes, mRNAs, and miRNAs provides insight into the regulatory potential of extracellular vesicles in recipient cell glucose metabolism

**DOI:** 10.3389/fbinf.2026.1911554

**Published:** 2026-08-07

**Authors:** Namita N. Kashyap, Sharath Mohan Bhat, Padmanabha Udupa E. G., Kavitha S. Shettigar, Vinutha R. Bhat, Dinesh Upadhya

**Affiliations:** 1 Department of Anatomy, Kasturba Medical College, Manipal Academy of Higher Education, Manipal, India; 2 Department of Ageing Research, Manipal School of Life Sciences, Manipal Academy of Higher Education, Manipal, Karnataka, India; 3 Department of Biochemistry, Kasturba Medical College, Manipal Academy of Higher Education, Manipal, India; 4 Department of Medical Laboratory Technology, Manipal College of Health Professions, Manipal Academy of Higher Education, Manipal, India

**Keywords:** extracellular vesicles, glucose metabolism, miRNA, molecular regulation, mRNA

## Abstract

**Introduction:**

Extracellular vesicles (EVs) are increasingly recognized as active coordinators of metabolic processes rather than mere messengers. By carrying unique subsets of enzymes, metabolites, lipids, and nucleic acids, EVs can directly deliver functional metabolic machinery or dynamically alter intracellular metabolic fluxes in recipient cells. However, their role in regulating specific biochemical pathways remains largely unknown.

**Methodology:**

In the current *in silico* analysis, we explored the dominant metabolic role of EV cargo using publicly available multi-omics data. For this, the top 500 mRNAs and proteins, along with miRNAs reported at least 10 times in humans across independent studies, as catalogued in the EVpedia database are considered and curated into a comprehensive dataset.

**Results:**

Enrichment analysis of these mRNAs and proteins revealed that carbohydrate metabolic pathways, including glycolysis, the pentose phosphate pathway and the TCA cycle, were over-represented in EVs. Further, to investigate whether EVs carry miRNAs that regulate these pathways, we analyzed the miRNA targets. Enrichment analysis of EV miRNA targets mapped glycolytic regulatory genes, including *HK1, HK2, PFKP and PKM*. Interestingly, we also found miRNAs targeting genes encoding glucose transporters (SLC2A1, SLC2A3, SLC2A4, and SLC2A14) reported in EVs. Genomic annotation of these miRNAs revealed them to form clusters, including the miR-17-92 cluster, a well-known regulator of glycolysis.

**Discussion:**

While the study has limitations—mainly due to the biological heterogeneity of EVs and the difficulty of standardizing cargo—the results potentially suggest that, by delivering enzymes, their mRNAs, regulatory miRNAs or combinations thereof, EVs could potentially mediate recipient cell glucose metabolism.

## Introduction

Extracellular vesicles (EVs) are the membrane-bound nanospheres released by cells that function as intercellular communicators in various physiological and pathological conditions. Although EVs are classified primarily into exosomes and microvesicles, the functional capacity of EVs as a whole remains unclear (Welsh et al., 2024). This is achieved through their cargo, which includes footprints of the parent cells, such as carbohydrates, lipids, proteins, nucleic acids and several metabolites, thus influencing the phenotype and molecular status of the target cell (Tkach and Théry, 2016). The selective packaging of these entities is a well-regulated, high-confidence process that elucidates the target response, thus establishing them as cellular signaling molecules (Dixson et al., 2023). To characterize the EV composition, multi-omics techniques have emerged as powerful tools, thereby enabling cargo investigation and establishing EVs as diagnostic, prognostic and therapeutic targets (Kalluri and LeBleu, 2020).

In the past decade, studies unraveling the metabolic landscape of EVs have gained momentum. EVs, as true representatives of their parental cells, shed light on the metabolic state of the parental cells. Numerous studies have explored the EV’s omics in diverse cell types, physiological and pathological conditions, including cancer, diabetes, hypertension, vascular dysfunction and more. For example, in carbohydrate metabolism, Guo et al. reported that exosomes derived from hepatocellular carcinoma cells carry CCT2, which, in M2 macrophages, stabilizes ALDOA, a key regulator of glycolysis (Guo et al., 2026). In high-fat diet (HFD)- fed mouse models, EVs derived from the gut microbiome of HFD-fed mice hampered the activation of AKT upon insulin treatment, indicating a possible mechanism of insulin resistance contributed by the gut microbiota (Choi et al., 2015). Another study by Cao et al. showed that breast cancer-derived EVs, through miR-122 targeting PKM, inhibited glycolysis in pancreatic beta cells (Cao et al., 2022). In another study, EVs derived from the blood of young participants were shown to target peroxisome proliferator-activated receptor (PPAR) γ coactivator α (PGC-1α), a master regulator of mitochondrial biogenesis and function, through their miRNA cargo (Chen et al., 2024). Miotto et al. reported that EVs derived from mouse liver enhanced blood glucose regulation by acting on peripheral tissues via endocrine signaling (Miotto et al., 2024). Ample research studies have demonstrated that EVs actively remodel the metabolic landscape of their surrounding microenvironment (Li et al., 2026; Puurand et al., 2025).

The clinical utility of EVs is becoming increasingly significant. The exponential growth in EV research has necessitated the development of databases to comprehensively catalog EV-associated molecules. Publicly available repositories such as Vesiclepedia, ExoCarta and EVpedia provide cumulative genomic, transcriptomic, proteomic and metabolomic data of EV cargo (Chitti et al., 2024; Kim et al., 2013; Keerthikumar, 2016). This vast repository of data enables large-scale *in silico* exploration, facilitating the identification of biomarkers and therapeutic targets across various physiological and pathological conditions. This also enables the cross-comparison and data-driven verification of published EV data with the meta-analysis.

In the present study, we sought to understand the functional landscape of EV cargo by curating a list of proteins, mRNAs and miRNAs from EVpedia. With the help of various bioinformatic tools and publicly available multi-omics datasets, we explored poorly understood biochemical pathways and molecular signatures in EVs.

## Methodology

### Data retrieval

The top 500 mRNAs and proteins were identified based on their detection frequency across independent studies and curated into a comprehensive dataset. To ensure reliability and reproducibility, all the data points were retrieved from the EVpedia database (updated in 2023, data retrieved on 04th March 2026). mRNA and protein subsets were standardized to official genomic nomenclature to reduce data redundancy, and overlapping entries were cross-verified. Duplicates between the subsets were removed to obtain a unified list of EV-associated genes.

### Pathway enrichment analysis

The consolidated gene list obtained from mRNA and protein data was functionally characterized using Kyoto Encyclopedia of Genes and Genomes (KEGG) enrichment analysis, which facilitated the identification of metabolic modules associated with EV cargo (Kanehisa et al., 2016). Enzyme-encoding genes were selectively extracted from this dataset to identify the metabolic routes. Functional annotation and hierarchical classification of the data set were performed using the KEGG BRITE functional hierarchy database. To determine over-represented biological classifications, a statistical enrichment analysis was performed using Fisher’s exact test to assess gene frequencies. Resulting p-values were adjusted using the Benjamini-Hochberg False Discovery Rate (FDR) method. BRITE modules were subsequently ranked based on a threshold of FDR <0.05.

To further understand the gene function, Gene Ontology (GO) enrichment analysis was performed using Enrichr, a cumulative analysis of all three major GO domains–biological process, molecular function and cellular component, which enabled the detailed examination of biochemical roles, enzymatic activities and subcellular localization patterns (Kuleshov et al., 2016). Statistical significance for each functional category was calculated by one-tailed Fisher’s exact test. Raw p-values were adjusted using the Benjamini-Hochberg FDR method. Final enriched pathways were ranked based on EnrichR’s combined score, which computes the product of the natural logarithm of the raw p-value and the z-score deviation from the expected rank (C = ln(p).z). Here, the macromolecular synthesis pathway was chosen to understand the highly regulated metabolic pathway via EVs. Furthermore, an overlap analysis between mRNAs and proteins was performed using KEGG to identify functional pathways represented at both levels in EVs.

### Mapping EV-associated miRNAs to their experimentally verified targets

miRNAs reported at least 10 times in EVpedia were listed. The targets of these miRNAs were identified using the miEAA tool (miRNA enrichment analysis and annotation, v 2.1). To execute this miRNA-centric approach, the standardized mature miRNA identifiers were synced to the miRBase database (v 22) (Chou et al., 2018). The miRNAs were subjected to over-representation analysis across curated functional categories, including all the reported target genes (miRTarBase v 10.0), KEGG pathways and disease associations. Fisher’s exact test was used to assess the statistical significance of the biological themes enriched by our input miRNAs relative to the universal miRNA reference background. P-values were adjusted using the Benjamini-Hochberg FDR method. Functional terms and downstream targets were considered significantly over-represented based on a threshold of FDR < 0.05. Only experimentally validated gene targets as reported in miRTarBase were considered for further analysis.

To complement the findings, genomic coordinates and chromosomal positions were retrieved through the BioMart interface in the Ensembl (release 115) genome browser. To evaluate regional localization patterns across the genome, a spatial density- and proximity-based clustering analysis was performed in R. Genomic regions were standardized to BED format, and clusters were defined using a sliding-window approach with a maximum inter-miRNA distance threshold of 10 kb. Genomic clusters containing fewer than two individual miRNAs were excluded from subsequent spatial filtering. To visually integrate these genomic features, a circular ideogram was constructed using the circlize framework in R, which allowed chromosomal clustering and mapping of functionally related families of miRNA targets of glycolysis (Kinsella et al., 2011). To identify miRNA clusters, a threshold of <10 kbp was set between the genomic coordinates.

### Visualization of data and statistical analysis

For data visualization and statistical analysis, R was used. Standardized graphical outputs, such as bar and bubble plots, for the enrichment analysis were generated using the ggplot2 package. To better visualize enrichment analysis graphs, Inkscape was used. Gene-miRNA interactions and chromosomal distributions were created using the circlize package (v 0.4.17) to obtain high-resolution representations of EV cargo. The R scripts used and the raw files curated for the analysis have been provided as Supplementary Material.

## Results

### EV cargo regulates macromolecular biosynthesis

**FIGURE 1:**
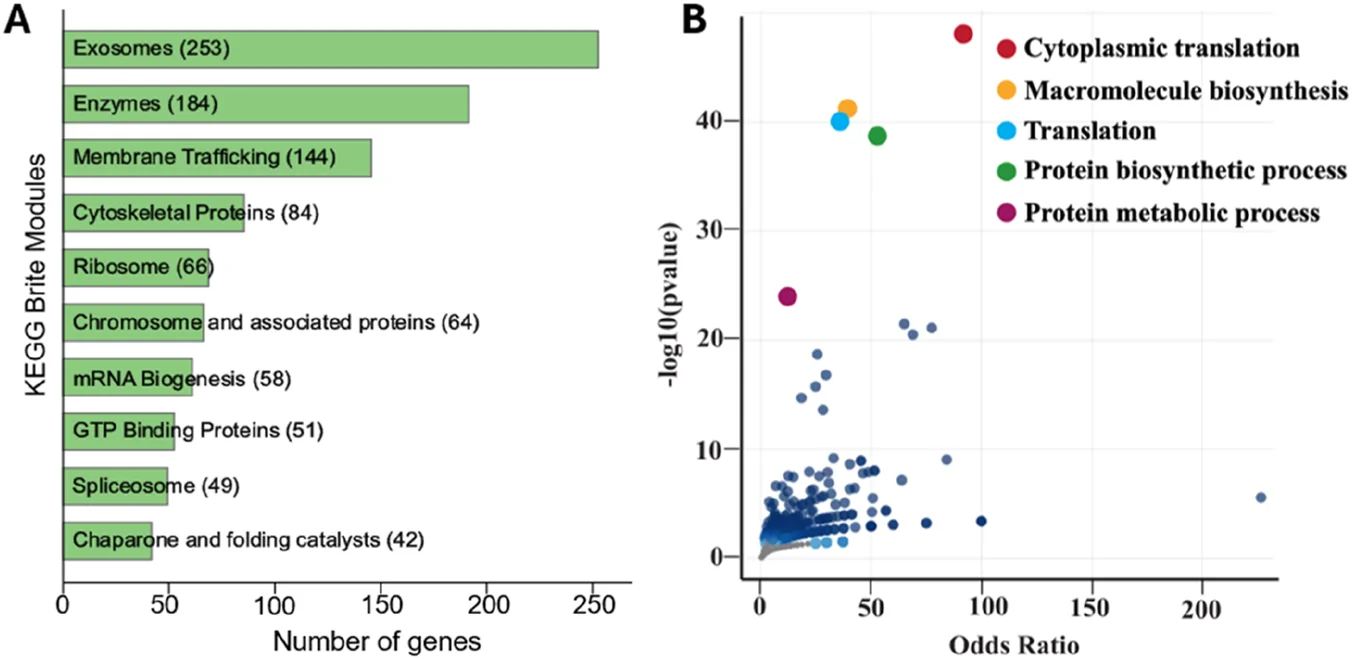
**(A)** The top 500 mRNAs and 500 proteins were subjected to an integrated analysis of gene enrichment, yielding the top 10 modules, which were visualized. As anticipated, 250+ genes associate with the exosomal module, followed by the enzyme-related category. **(B)** Further enrichment analysis of common genes between the two categories for biological processes yielded the indicated top 5 pathways. Analysis was performed using EnrichR. To enhance interpretability, the graphs were edited in Inkscape. The statistical test performed was Fisher’s exact test. Post hoc analysis was performed using the Benjamini-Hochberg FDR method. p < 0.05 was considered statistically significant.

A list of the top 500 mRNAs and top 500 proteins most frequently reported in EVs of diverse origin was compiled from EVpedia. A list of 858 genes (after removing duplicates) was further processed to understand the functional landscape of EVs. To better understand the macromolecular biosynthetic processes occurring in EVs, we investigated these genes within KEGG BRITE modules (Figure 1A). Here, we identified that 184 of these genes were enzymes. Interestingly, we found several metabolic enzymes such as *ENO1, GAPDH, PKM, ALDOA, GPI, PFKL, PFKM, PGAM1, PGK1* and *TPI1,* which are involved in glucose metabolism, including glycolysis and gluconeogenesis, *G6PD*, *PGLS, GPI, PGD, TALDO1, TKT,* which are involved in the pentose phosphate pathway, *CS, IDH1, IDH3B, MDH1, MDH2* and *SDHC,* which are involved in the citric acid cycle. We also found *FASN, TECR* (involved in fatty acid biosynthesis), DUT, NME1, PAICS, ATIC, CMPK1, PNP, NT5E and IMPDH2, which are involved in nucleic acid metabolism. About 30% of the genes (141) were reported to be expressed at both the mRNA and protein levels, indicating multifactorial regulation of diverse processes (Figure 1B). Enrichment analysis of these genes for biological processes indicated that they were involved in cytoplasmic translation, macromolecular biosynthesis and protein metabolism. This analysis indicated that EV cargo is rich in mRNAs and proteins that regulate macromolecular metabolism, including carbohydrate, lipid and nucleic acid metabolism (Supplementary Table S1).

**FIGURE 2:**
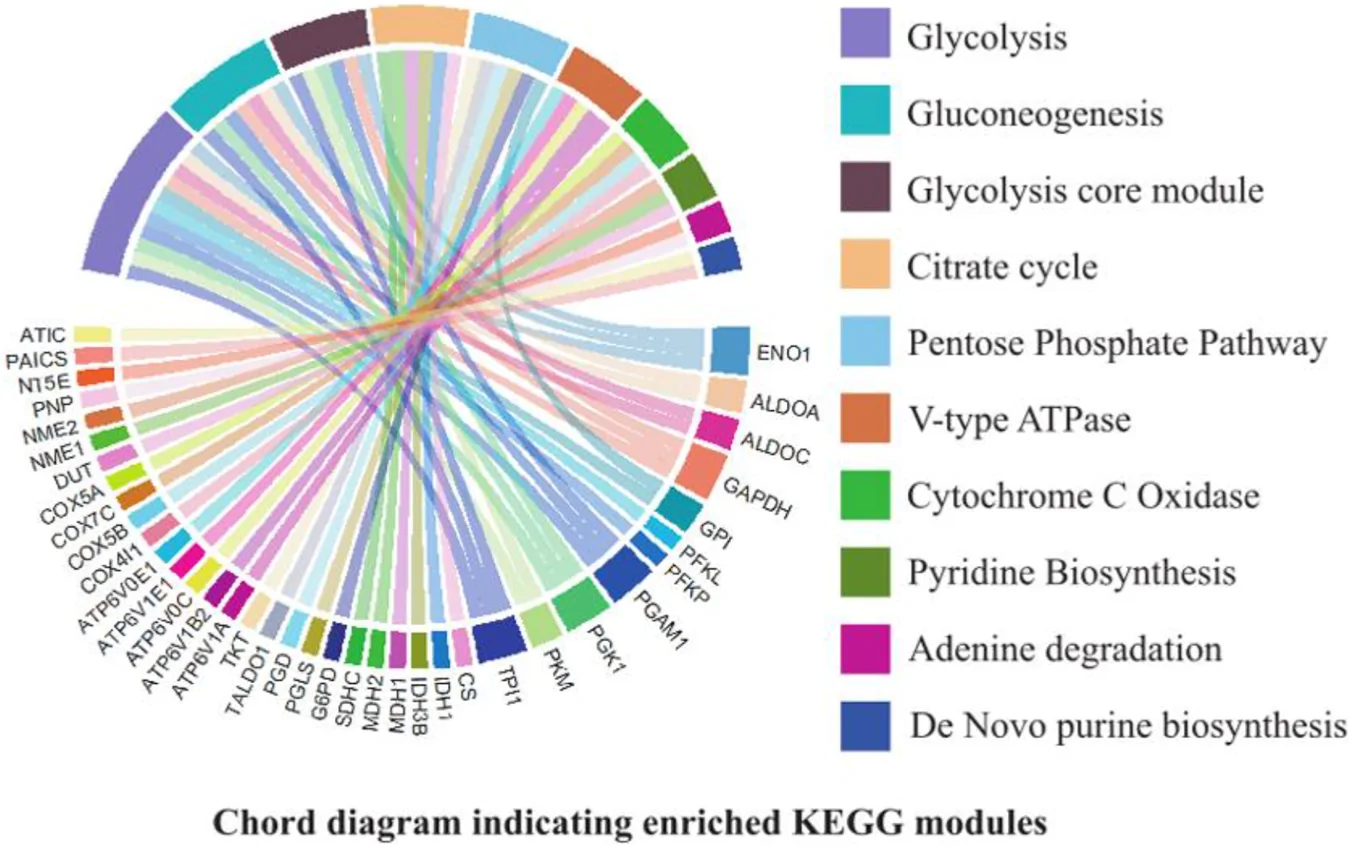
Investigation of the macromolecular biosynthetic pathways associated with exosomal enzymes revealed all the top 5 enriched pathways to be related to glucose metabolism. The R package circlize was used for image generation.

To further explore the macromolecular biosynthetic processes, we performed KEGG MODULE enrichment analysis of the gene list. We found that glucose metabolic pathways, including glycolysis, gluconeogenesis and the pentose phosphate pathway, were overrepresented in EVs. Also, we found nucleic acid metabolic pathways such as *de novo* purine biosynthesis, pyridine biosynthesis and adenine degradation to be overrepresented in EVs (Figure 2).

**FIGURE 3:**
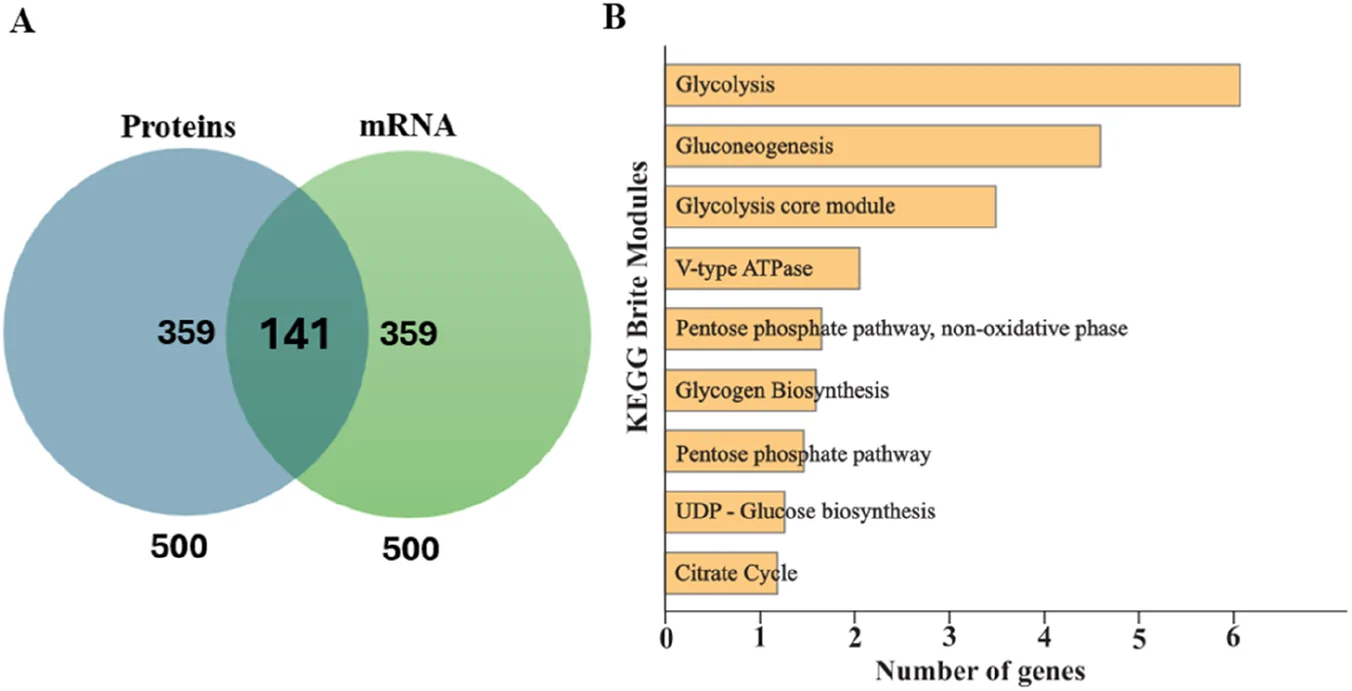
**(A)** The Venn diagram illustrates the overlapped genes between the proteins (n = 500) and mRNA (n = 500). A total of 141 common genes were identified. **(B)** The identified common genes were analyzed for pathway enrichment, revealing that 4 of the top 5 enriched pathways are related to glucose metabolism. Analysis was performed using KEGG. To enhance interpretability, the graphs were edited in Inkscape. The statistical test performed was Fisher’s exact test. Post hoc analysis was performed using the Benjamini-Hochberg FDR method. p < 0.05 was considered statistically significant.

141 genes were found to overlap between mRNA and protein levels (Figure 3A). Enrichment analysis of the overlapping genes between mRNA and protein was performed. Glycolysis, gluconeogenesis and other glucose-related pathways emerged as the top pathways when subjected to KEGG brite modules (Figure 3B).

**FIGURE 4:**
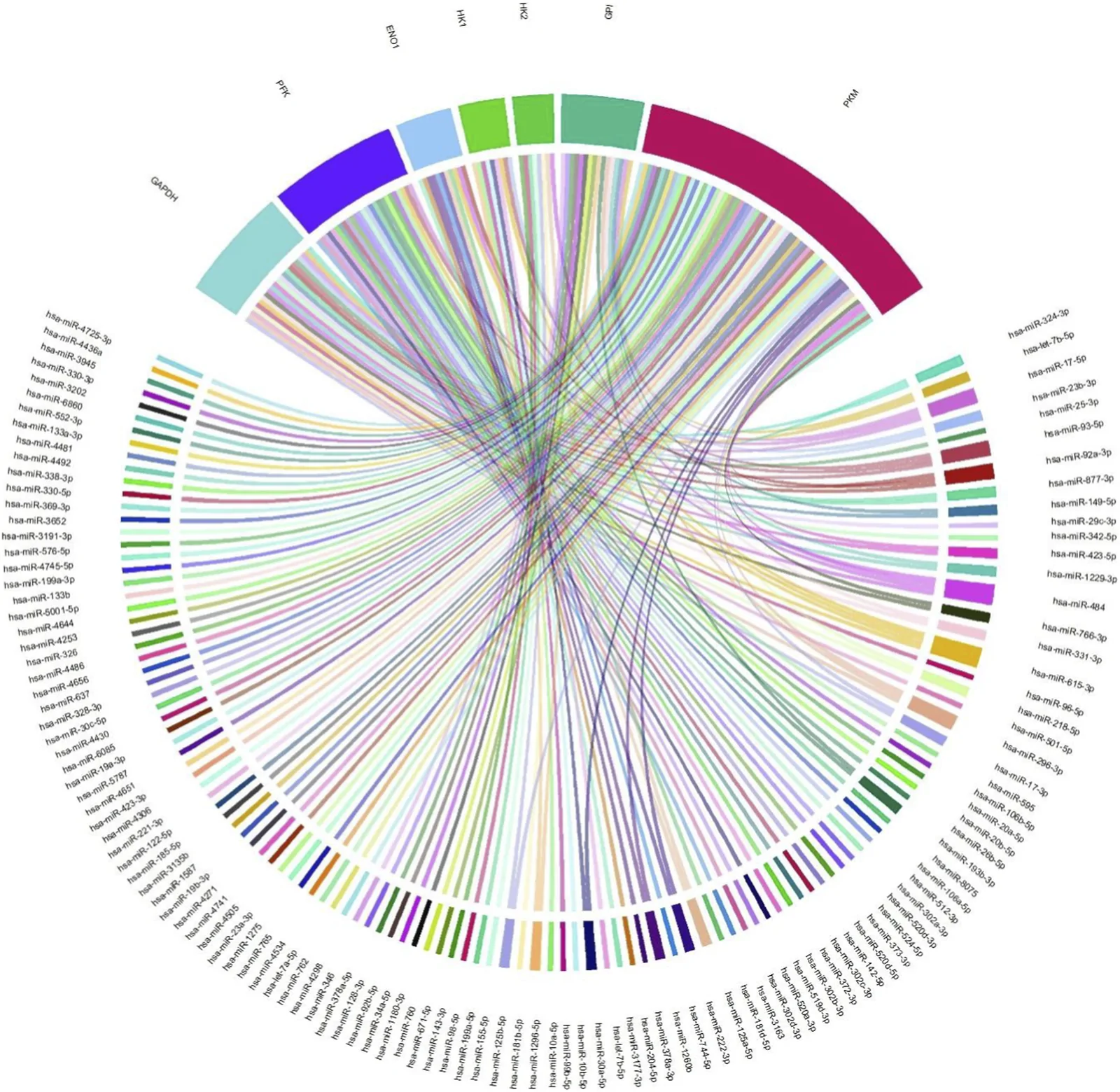
miRNAs reported at least 10 times in EVpedia (n = 757) were selected for analysis. The predicted targets of these miRNAs were subsequently identified (n = 2316) using miRTarBAse, which were further subjected to pathway enrichment analysis. Glycolysis emerged as the most significantly miRNA-enriched pathway, yielding seven genes associated with enzymes of the glycolysis pathway. The above chord diagram was constructed to map miRNA-gene interactions. Notably, all three rate-limiting enzymes of glycolysis were identified as targets of these miRNAs. Genes of glycolysis identified as miRNA-enriched targets–GAPDH, PFK, ENO1, HK1, HK2, GPI, PKM. R package - circlize was used to plot the chord diagram.

To further explore if there are miRNAs present in the EVs that contribute to macromolecular metabolism, we made a list of 757 miRNAs that have been reported in EVs in at least 10 independent studies. Targets of these miRNAs were identified using the miRTarBase database. A total of 2,316 targets were identified. Interestingly, we found several miRNAs targeting glycolytic genes. There were 22 miRNAs predicted to target GAPDH, including hsa-miR-25-3p, hsa-miR-93-5p, hsa-miR-92a-3p, hsa-miR-877-3p and more. There were 25 miRNAs predicted to target PFKP, including hsa-miR-595, hsa-miR-17-5p, hsa-miR-106b-5p, hsa-miR-93-5p, hsa-miR-20a-5p, hsa-miR-20b-5p and more. 11 miRNAs including hsa-miR-181d-5p, hsa-miR-92a-3p, hsa-miR-125a-5p, hsa-miR-222-3p targeting ENO1. 9 miRNAs targeting HK1, 8 miRNAs targeting HK2 and 16 miRNAs targeting GPI. Interestingly, we identified 64 miRNAs targeting PKM. This included miRNAs such as hsa-miR-324-3p, hsa-miR-4298, hsa-miR-762, hsa-let-7a-5p, hsa-miR-4534 (Figure 4).

Further, to explore if these miRNAs form clusters and are possibly regulated by polycistronic transcription, a threshold of <10 kb was set as a distance criterion. Notably, significant enrichment of miRNA clusters targeting these genes were observed, signifying that EVs are selectively enriched with miRNAs involved in glycolytic regulation.

**FIGURE 5:**
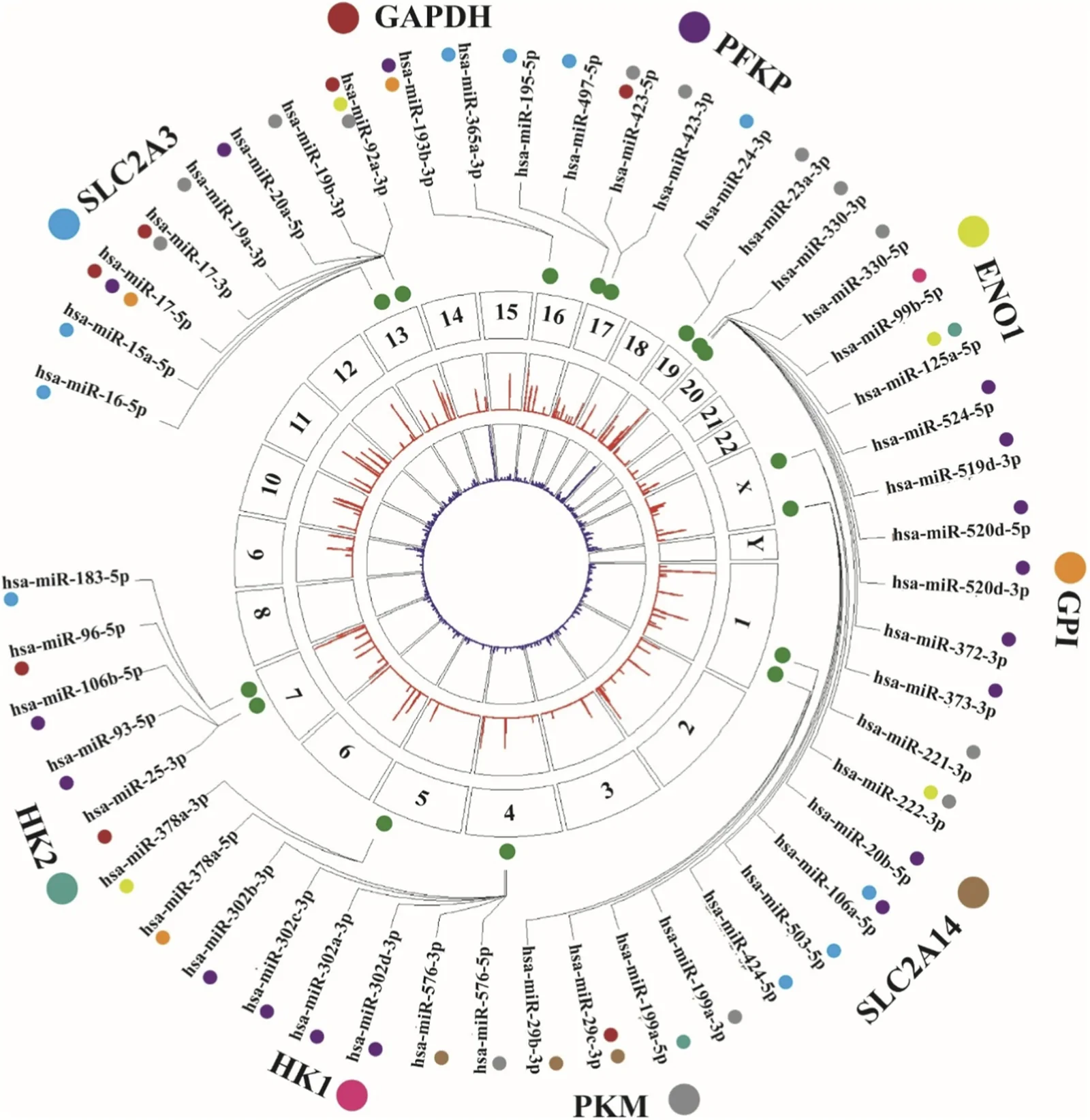
The inner genomic density plot (purple track) depicts the miRNAs that were identified at least ten times in the EVpedia database, indicating high-confidence and frequently reported miRNA species. The subsequent enrichment track (orange) highlights the genomic distribution of miRNAs targeting glycolytic genes identified in Figure 4, as well as glucose transporter genes. The numbers 1–22 with X and Y indicate chromosomes. The subsequent track indicates miRNA clusters obtained using miRTarBase. The R package circlize was used to visualize the data points.

Furthermore, to identify the glycolytic regulatory milieu of EVs, we assessed whether any of these miRNAs target glucose transporters. Interestingly, we found 14 miRNAs targeting SLC2A1, 24 miRNAs targeting SLC2A3, 8 miRNAs targeting SLC2A4 and 13 miRNAs targeting SLC2A14. This indicated that the EVs finely tuned glucose transporter regulation. Since many miRNAs appear as miRNA clusters in the genome, we mapped the genomic location of these miRNAs. We identified miRNAs from multiple clusters, including the well-known miR-19-72 cluster, which has been established to regulate glycolysis in multiple cell types (Figure 5).

## Discussion

This integrated analysis of proteins, mRNA and miRNA associated with EVs supports the conclusion that EVs significantly contribute to the regulation of glycolysis in recipient cells. This convergence across three independent molecular subsets suggests that cargo sorting into EVs is rather a tightly regulated, highly coordinated process than a random assortment of cell components. The presence of glycolytic enzymes, their transcripts, and miRNA clusters elucidates a goal-directed packaging of the metabolic machinery into EVs, suggesting a role for EVs in metabolic regulation.

The earliest studies have established that the recipient cell metabolism could be modified by the transfer of EV components (Valadi et al., 2007). The consistent presence of glycolytic enzymes and mRNAs in EVs enables EVs to adapt to diverse physiological conditions by promoting glycolytic flux in recipient cells. In glycolytic-dominant metabolic reprogramming via tumor-derived EVs, macrophages have been polarized toward an immunosuppressive phenotype (Morrissey et al., 2021). These studies highlight the regulatory role of enzymes and specific proteins in EVs in modulating glycolysis.

The presence of large amounts of mRNA and other small RNAs in EVs is not due to passive loading but is rather a selective process (Guduric-Fuchs et al., 2012). Studies demonstrated that mRNA delivered from EVs into recipient cells generates a functional protein (Skog et al., 2008; Zomer et al., 2015). This suggests that specific mRNAs and miRNAs might have been loaded into EVs for their functional utility in recipient cells. The microRNAs are small endogenous noncoding RNAs (Lau et al., 2001) that possess the ability to bind to different regions of mRNA, such as the 5′-UTR and protein-coding exon regions, but the base pairing between the miRNA seed region and the 3′-UTR of mRNA is mainly responsible for the quantitative regulation of mRNAs or their protein forms (Lewis et al., 2005). The presence of miRNAs associated with glucose metabolism corroborates the caliber of EVs in not only delivering transcriptional and translational modules but also post-transcriptional regulators that can possibly modulate glycolytic flux.

While this study provides interesting insights into EV-mediated metabolic targeting, it has certain limitations. The current analysis relies on data curated from EVpedia, which contains cumulative reports from diverse EV sources with varying isolation methods, leading to variability across EV populations (Welsh et al., 2024). For the present study, due to limitations in the classification of EVs in the EVpedia database, we have treated EVs as whole entities rather than as exosomes or microvesicles. Also, we could not perform EV cargo normalization due to high biological heterogeneity and the lack of universal reference standards, which might have influenced study outcomes. In addition, for the current analysis, the top 500 proteins and mRNAs were considered to reduce study noise and capture the core set of frequently reported molecules. To reiterate that we have not overlooked any important molecules, we have performed the same analysis using the top 1,000 mRNAs and proteins from EVpedia. Both analyses yielded similar data with no significant alterations to our interpretations (Supplementary Figures SF1–SF3).

Furthermore, this paper acknowledges the presence of various molecular components but does not experimentally validate their functional activity. Also, miRNA targets and clustering are based solely on a few previously published datasets, which may affect the results. These limitations could pave the way for new prospects in the field. With recent breakthroughs in EVs, the need for a database that selectively classifies EVs by source and isolation technique is significant. Additionally, the practical implications of the aforementioned findings could significantly enhance understanding of EVs.

In summary, while EV source variability and cargo normalization are limitations, the presented data demonstrate coordinated enrichment of glycolytic enzymes, transcripts, and miRNA regulators in EVs, underscoring an effective, robust and possible functional role of EVs in recipient cell glucose metabolism.

## Data Availability

The original contributions presented in the study are included in the article/Supplementary Material, further inquiries can be directed to the corresponding author.
